# Waves of layered immunity over innate lymphoid cells

**DOI:** 10.3389/fimmu.2022.957711

**Published:** 2022-10-04

**Authors:** Toshiaki Kogame, Gyohei Egawa, Takashi Nomura, Kenji Kabashima

**Affiliations:** Department of Dermatology, Graduate School of Medicine, Kyoto University, Kyoto, Japan

**Keywords:** ILC, layered immunity, prenatal development, thymic origin, heterogeneity

## Abstract

Innate lymphoid cells (ILCs) harbor tissue-resident properties in border zones, such as the mucosal membranes and the skin. ILCs exert a wide range of biological functions, including inflammatory response, maintenance of tissue homeostasis, and metabolism. Since its discovery, tremendous effort has been made to clarify the nature of ILCs, and scientific progress revealed that progenitor cells of ILC can produce ILC subsets that are functionally reminiscent of T-cell subsets such as Th1, Th2, and Th17. Thus, now it comes to the notion that ILC progenitors are considered an innate version of naïve T cells. Another important discovery was that ILC progenitors in the different tissues undergo different modes of differentiation pathways. Furthermore, during the embryonic phase, progenitor cells in different developmental chronologies give rise to the unique spectra of immune cells and cause a wave to replenish the immune cells in tissues. This observation leads to the concept of layered immunity, which explains the ontology of some cell populations, such as B-1a cells, γδ T cells, and tissue-resident macrophages. Thus, recent reports in ILC biology posed a possibility that the concept of layered immunity might disentangle the complexity of ILC heterogeneity. In this review, we compare ILC ontogeny in the bone marrow with those of embryonic tissues, such as the fetal liver and embryonic thymus, to disentangle ILC heterogeneity in light of layered immunity.

## Introduction to ILC biology

The terminology innate lymphoid cells (ILCs) includes a wide range of cell types, such as natural killer (NK) cells, which were discovered in 1975 and has a damaging activity against virus-infected cells and tumors (1), and lymphoid tissue inducer (LTi) cells, which were discovered in 1997 and play essential roles in the formation of secondary lymphoid tissue, such as Peyer’s patch (2), ILC3 which serves for innate mucosal immune defense with Th17 cytokine production such as IL-22 (3–6), and non-T/non-B cells that induce type 2 responses in an IL-25-dependent manner, which was reported in 2001 (7) and turned out to play a pivotal role in allergic conditions by producing Th2 cytokines in 2010 (8–10). Thus, various names had been assigned to these cells during decades of ILC history. The nomenclature and terminology regarding mouse and human ILCs have recently been updated (11) and now include five groups: cytotoxic NK cells, group 1, 2, and 3 innate lymphoid cells (ILC1, ILC2, and ILC3), and LTi cells. ILC1 produce type 1 cytokines (12). ILC2 is characterized by the expression of GATA-3, a master regulator of Th2 differentiation, and secretes type 2 cytokines, such as IL-5 and IL-13 (13). The expression of RORγt, a critical transcriptional factor for Th17 differentiation, allows ILC3s to produce Th17-related cytokines IL-17A and IL-22 (14). Murine experiments indicated that LTi cells similarly express RORγt in addition to AHR, RUNX3, Notch, and Arg1 to promote secondary lymphoid tissue formation prenatally (11, 15–19). NK cells harbor similar characteristics to ILC1 but, unlike ILC1, express granzymes and perforin and has cytolytic activity. These characteristics make them analogous to T-cell subsets, such as CD4^+^ Th1 cells with ILC1, CD4^+^ Th2 cells with ILC2, ILC3 with Th17 cells, and NK cells with CD8^+^ cytotoxic T cells. ILCs harbor the characteristic nature of the tissue residency. The parabiosis experiment with mice demonstrated that most ILCs were not replenished by blood supply (20). Other unique characteristics include unstable and elusive phenotypes, e.g., ILC1 can convert into ILC3 or vice versa (21). Furthermore, phenotypes of ILCs also differ according to anatomical site, e.g., the phenotypical difference between lung ILC2 and skin ILC2 (22). These characteristics of ILCs synergistically result in the significant heterogeneity in ILCs and bring a daunting difficulty in understanding the whole picture of ILC biology [for review (23)]. However, recent scientific progress provides ILC biology with an aspect of developmental biology. Here, we update ILC ontology in different niche sites. In addition, we introduce the concept of “layered immunity” which helps us to comprehend the heterogeneity of ILC biology.

## Variation of the ontogeny of ILCs

**Figure 1:**
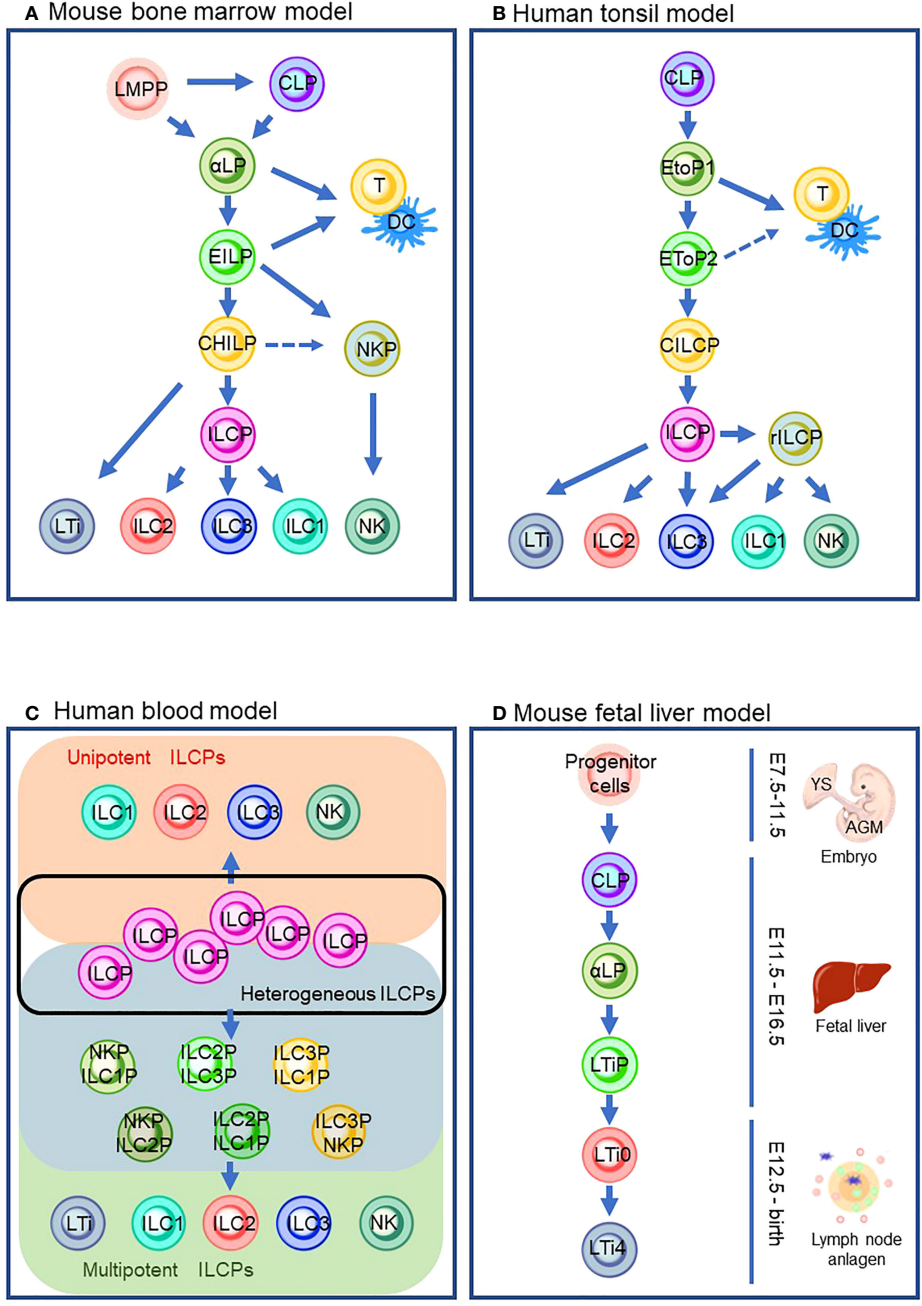
ILC differentiation pathways in the different models that are currently proposed. **(A)** Mouse bone marrow model. **(B)** Human tonsil model. **(C)** Human blood model which describes the heterogeneous ILCP group (inside a black line), ILC subsets from unipotent ILCPs (orange background), and ILC subsets from multipotent ILCPs (green background) *via* its intermediates (blue background). **(D)** Mouse fetal liver model. Solid lines indicate the differentiation pathway which is considered the main pathway. Dashed lines indicate the alternative pathways that are demonstrated by the experiments in specific conditions. LMPP: lymphoid primed multipotent progenitors; CLP, common lymphoid progenitor; αLP, α-lymphoid precursor cells; EILP, early inmate lymphoid progenitor; DC, dendritic cells; CHILP, common helper-like innate lymphoid progenitor; NKP, natural killer cell progenitor; ILCP, innate lymphoid cell progenitor; ILC1/2/3, group 1/2/3 innate lymphoid cell; NK, natural killer cell; LTi, lymphoid tissue inducer; EToP1/2, early tonsil progenitor 1/2; CILCP, common innate lymphoid cell progenitor; YS, yolk sac; AGM, aorta–gonad–mesonephros.

Since progenitor cells of ILCs were discovered in mouse bone marrow (BM) (24), ILC ontology has been intensively studied with BM in adult mice (**Figure 1A**). Mouse ILC ontogeny originates from common lymphocyte progenitor cells (CLPs), which can give rise to all lymphocyte subsets, including ILCs (25). Nfil3 is a basic leucine zipper transcription factor essential for ILC lineage commitment (26, 27). Previous studies showed that mice lacking Nfil3 were deficient in all mature ILCs, including NK cells (26, 28). By genome analysis of *in vivo*-derived cells, α-lymphoid precursor cells (αLPs), which were defined as CXCR6^+^α4β7^+^IL-7R^+^ cells, were demonstrated to differentiate from mouse CLPs (27, 29). A phenotypically functionally combined approach with fate mapping with the Tcf7 gene, a T cell-specific transcription factor downstream of Notch/Wnt signaling (30, 31), showed that early innate lymphoid progenitors (EILPs) were also differentiated from CLPs (32). A recent study clarified the relationship between αLPs and EILPs (32). In the study, a reporter mouse system expressing Tcf7^EGFP^ in addition to fate-mapped CD127^YFP^ were used to demonstrate that GFP^+^ EILPs were CD127 fate map positive. This observation suggested that EILPs reside downstream of αLP (33). It is of note that differentiation from CLPs toward EILPs *via* αLPs requires TOX, a transcriptional factor essential for the CD4 lineage program, since TOX regulates the gene expression of Tcf7 (34, 35). A reporter mouse experiment for Id2, a central hub in controlling helper-like ILC identity, showed that ILCs and NK cells differentiate from EILPs *via* branching to common helper-like ILC progenitors (CHILPs) and NK progenitors (NKPs) (32, 36–40). EILPs also harbor the potential to differentiate into dendritic cells (DCs) (41). CHILPs are divided into two groups depending on the expression of a transcription factor, promyelocytic leukemia zinc ﬁnger protein (PLZF which is coded in the Zbtb16 gene), which induces the maturation of various ILC subsets such as ILC2, ILC3, and some ILC1, but not NK cells or LTi cells (42–44). The PLZF^+^ group called ILC precursors (ILCPs) differentiate into NK cells and ILC1/2/3s that are characterized by the expression of T-bet, GATA-3, or RORγt, respectively (43, 45, 46). Contrarily, the PLZF^-^ population differentiates into LTi-like ILC3 (43, 45). Furthermore, CHILPs also retained NK potential (45). By definition, CHILPs generate only helper-like subsets of ILCs but not NK cells. Thus, CHILPs with NK potential need further study for proper categorization in the developmental path. It is of note that lymphoid-primed multipotent progenitors (LMPPs) serve as the progenitor of CLP in the BM. However, a recent study suggested that LMPPs bypass CLP to generate downstream lineages (24, 47). Thus, it is implied that ILC ontogeny may differ according to spatiotemporal context.

Although it is known that BM seeds ILCs into peripheral tissue *via* circulation, most ILCs are long-lived and tissue-resident with minimal turnover (48). Thus, ILCs are considered to differentiate and propagate in the niches of various tissues. In addition, it is yet to be fully clarified how circulating ILCs contribute to tissue-resident ILCs. Human ILCs are most studied in the tonsil (**Figure 1B**). It is of note that the similarity between mouse and human ILC developmental pathways is observed (**Figures 1A, B**). Their development begins with CLP (49). CLPs differentiate into early tonsillar progenitors (EToPs) which are subdivided into two groups, EtoP1 (defined as Lin^-^CD34^+^CD10^+^KIT^-^) then EtoP2 (defined as Lin^-^CD34^+^CD10^-^KIT^+^) (50, 51). Both populations are multipotent and can generate T cells and DCs *in vitro* (52). IL-1R1 expression further subdivides the EtoP2 population (52). IL-1R1^-^ EtoP2 differentiates into not only mature ILCs but also T cells and DCs (52). In contrast, IL-1R1^+^ EtoP2 cells are known to differentiate exclusively toward ILCs and are considered as committed common innate lymphoid progenitors (CILCPs) (24). CILCPs with limited differentiation potential, defined as Lin^-^CD34^-^CD7^+^IL-7R^+^KIT^+^ cells, were found to differentiate exclusively into ILCs in various tissues (53). Thus, this cell type was annotated as ILCPs in human. Downstream of ILCPs, CD34^-^KIT^+^CD56^+^ cells, which are called restricted ILCPs (rILCPs), encompass a further restricted differentiation potential (52). NK cells, ILC1/3s, but not ILC2, were produced from rILCPs (54).

Since a unique ILC population with CD127^+^CD117^+^ phenotype resembles tonsil ILC3, the cells with this phenotype circulating in the human blood have long been regarded as ILC3 (55). Nevertheless, in the recent report, CD7^+^CD127^+^CD117^+^ cells in the peripheral blood which resemble tonsil ILC3 were demonstrated to express IL-17A and RORγt only at the traceable level (53). An *in vitro* culture experiment showed that the cells generated ILC subsets, including NK cells, but not other hematopoietic lineages. The single-cell RNA sequence (scRNA-sec) showed that human ILCPs are a heterogeneous population that was composed of multipotent and unipotent ILCPs (**Figure 1C**). Thus, CD7^+^CD127^+^CD117^+^ cells are regarded as a circulating ILC subset with characteristics of human ILCPs. The group also discovered that IL-1β is a potent growth factor for ILCs and triggered ILCP development along with IL-2 and IL-7 *in vitro* (56). The requirement of IL-1β in human ILCP differentiation leads to a stepwise model of ILC development in peripheral tissue: (1) circulation of ILCPs in the blood, (2) ILCPs’ response to increased IL-1β in inflamed tissue, (3) locoregional proliferation of ILCPs, and (4) differentiation into mature ILC subsets (53, 57, 58). It is suggested that the blood-circulating ILCPs could provide “on-demand” replenishment of ILC subsets in the inflamed sites as their niche to differentiate and propagate, which makes a clear contrast to naïve T cells in the lymph node (LN). However, ILCPs harbor differentiation potential into ILC subsets (e.g., ILC1/2/3 with T-bet/Gata-3/RORγt expression functionally reminiscent of Th1/2/17) and a quiescent state with reduced glycolysis and mitochondrial activity, without effector cytokine production or extensive proliferation, which all associate with the characteristics of naïve T cells (59). This tempts us to speculate that the biological significance of the blood-circulating ILCPs may be an innate counterpart of naïve T cells, which differentiate into helper and cytotoxic T cells of the acquired immune system.

LTi cells can be produced postnatally in ILC ontogeny in mouse BM (60, 61) (**Figure 1A**). However, much effort has been made to clarify the embryonic ontogeny of LTi cells since LTi cells are associated with the formation of the secondary lymphoid organs in the embryonic phase (60). Previous studies revealed that the fetal liver (FL) serves as a niche for ILC development. The extra-embryonic yolk sac (YS) and aorta–gonad–mesonephros (AGM), an intra-embryonic hematopoietic site derived from methoderm, are known to produce hematopoietic progenitors during embryonic gestation days 7.5 (E7.5)–E9 and E8.5–E11.5, respectively (62–64) (details are described in Section 5). Hematopoietic progenitors from YS and AGM migrate toward FL around E12.5 (65) and differentiate into CLPs (66). Subsequently, CLPs differentiate into αLPs which express Id2. Id2 is known to repress E2A, a member of the E protein family which transcriptionally regulates many developmental processes, including B- and T-cell differentiation (67). Notably, a recent study demonstrated that Id2 prevents chromatin remodeling to differentiate toward naïve CD8 T cells, implying that Id2 might maintain the chromatin state essential for ILC lineages (68). Instead of expressing PLZF to differentiate into ILCPs, αLPs in FL differentiate into LTi precursors (LTiPs) that express CXCR5 and CXCR6, in addition to Tcf7 (69). These chemokine receptors expressed in LTiPs facilitate migration to the LN anlagen, where the expression of RORγt triggers further differentiation into mature LTi4 which expresses LTi potential marker, CD4 *via* CD4^-^ LTi0 (33, 70).

Although the variation of ILC ontogeny between human and mouse may be due to the difference in species, the ontogenic variation among tissues in the same species may suggest that hematopoietic progenitors in different niches can produce distinct ILC subsets according to locoregional biological processes. In the following sections, we summarize experimental findings about ILCs in the embryonic thymus as an example of another prenatal ontogeny of ILCs. Furthermore, we introduce the concept of layered immunity to explain the phenomena where phenotypically different ILC subsets collectively exert robust immunity.

## Embryonic development of T-cell lineage in the thymus

**Figure 2:**
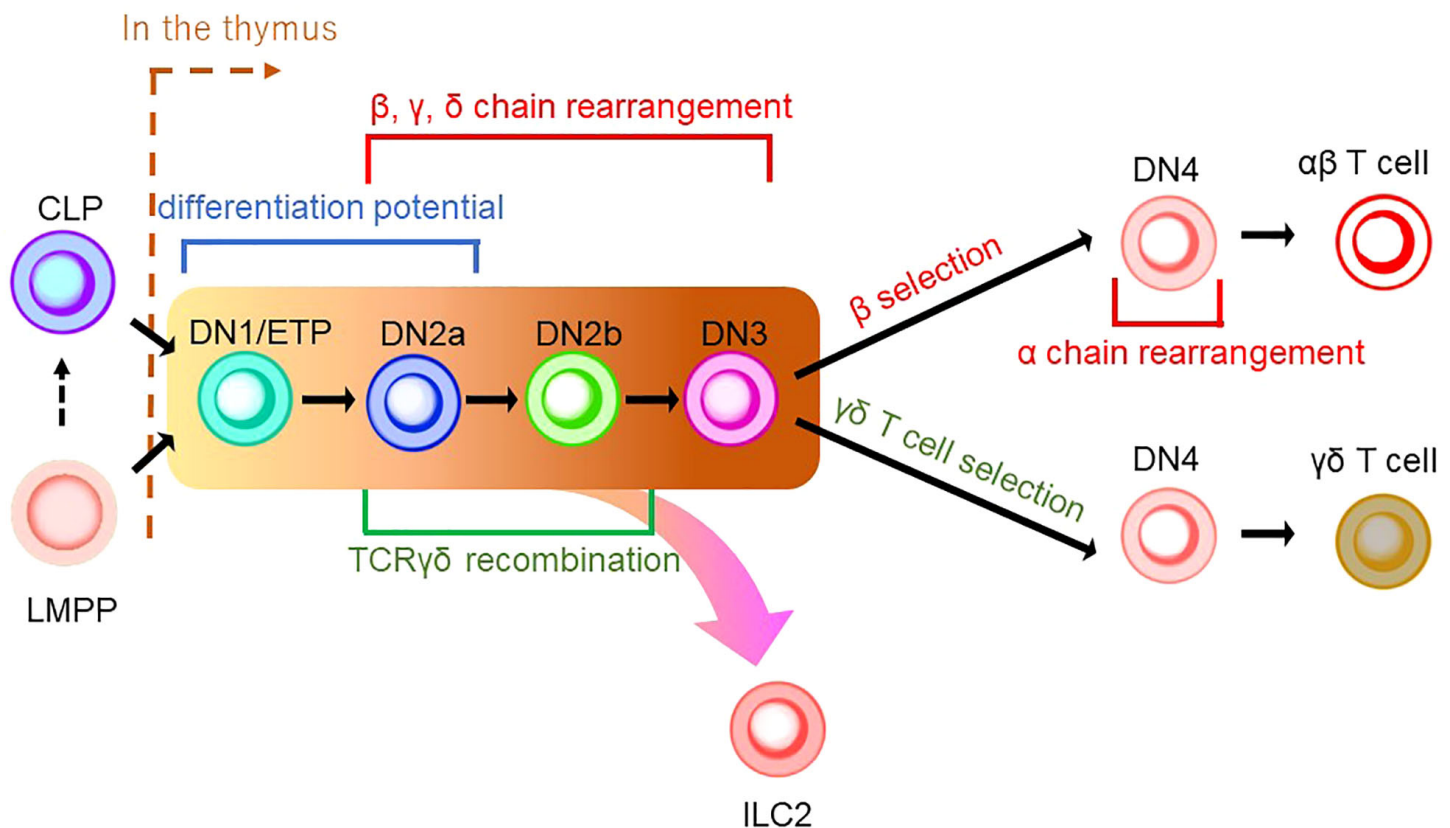
A schematic model of thymic development of T-cell lineages. CLP or LMPP is seeded into the thymus to become a thymocyte (the right side of a brown dashed line indicates thymic development). Thymocyte develops from a series of double-negative (DN) cells. αβ T cells and γδ T cells share the same developmental pathway from DN1/ETP to DN3 (indicated by the background in yellow to brown). DN1/ETP and DN2a harbor the differentiation potential as EILP in mice and EToP1/2 in humans (see **Figure 1**). T-cell receptor (TCR) recombination occurs during DN cell phases. Specifically, DN2a/b and DN3 undergo recombination of β, γ, and δ chains, while DN4 cells in the β-selection pathway do that of the α chain. TCRγδ recombination is known to occur in the phase between DN2a/b. In DN4 stage, γδ T-cell selection induces γδ T-cell development, while β-selection leads to αβ T-cell differentiation. ILC2 may be derived from the transition between the DN1/early thymic progenitor (ETP) and DN3 in the γδ T-cell development pathway.

As described in the previous section, ILCPs were discovered in BM. Furthermore, early studies characterizing ILCs with Rag-deficient mice demonstrated that ILCs could be generated without Rag expression, raising the possibility that ILCs are not of the T-cell lineage (71). Thus, the association of ILCs to the T-cell lineage had been overlooked. Nonetheless, in addition to the resemblance of ILCPs to naïve T cells in recent studies, we discuss in the following section that the T-cell developmental pathway in the thymus plays an essential role in ILC ontology. T-cell differentiation is unique compared to other hematopoietic lineages since it requires the maturation process in the thymus before its colonization into peripheral tissues. The differentiation pathway of the T-cell lineage is commenced from either CLPs or LMPPs (**Figure 2**) (72–74). Upon reaching the thymus, CD4^-^CD8^-^ thymus seeding progenitors (TSPs) enter the double-negative (DN) phase of T-cell differentiation. The developmental phases of DN thymocytes are classified as DN1/early thymic precursor (ETP), DN2a, DN2b, DN3, and DN4 according to expression levels of CD24, CD25, CD44, and KIT and the status of the TCR reconstitution (72, 73). DN1 cells have the ability and pluripotency to differentiate into myeloid and lymphoid lineages. However, the Notch-induced genetic program is activated to induce differentiation toward the T-cell lineage after the migration of DN1 cells into the corticomedullary junction (72, 73). As they progress to the DN2 stage, they localize to the subcapsular zone of the thymic cortex. They initiate the site-specific recombination of TCRβ, γ, and δ loci *via* Rag1/2 which cleaves DNA at conserved recombination signal sequences of the TCR locus (75). The proliferation and differentiation of DN2 require IL-7 (76). DN2 is subdivided into DN2a and DN2b according to the status of lymphocyte-specific protein tyrosine kinase (Lck) expression and differentiation potentials to NK cells, myeloid cells, and DC progenitors. It is of note that the differentiation potential toward DC, which EILPs in mouse and EToP1/2s in human harbor, is seen in DN1/ETPs and DN2a cells, but not in DN2b cells (**Figures 1A, B** and **2**) (77). In the DN3 population, only cells expressing functional γδ or preTα/β (preTCR) chains go on to survive after the rearrangement of the TCR β, γ, and δ loci. The generation of a functional TCRβ chain serves as a checkpoint, which is termed “β-selection”. A successful rearrangement of the TCRβ chain leads to preTCR formation (78). PreTCR^+^ DN3 moves to the DN4 stage, where thymocytes return to the medulla and initiate TCRα gene reorganization by preTCR signaling. After expressing functional CD4 and CD8 receptors, thymocytes become double-positive (DP) thymocytes. Then, DP cells are positively selected for reactivity with MHC, resulting in CD4 or CD8 single-positive (SP) cells. Subsequently, the surviving SP cells undergo negative selection with autoreactivity to become mature naive T cells as a consequence of the αβ T-cell developmental pathway (**Figure 2**) (72, 73).

**Figure 3:**
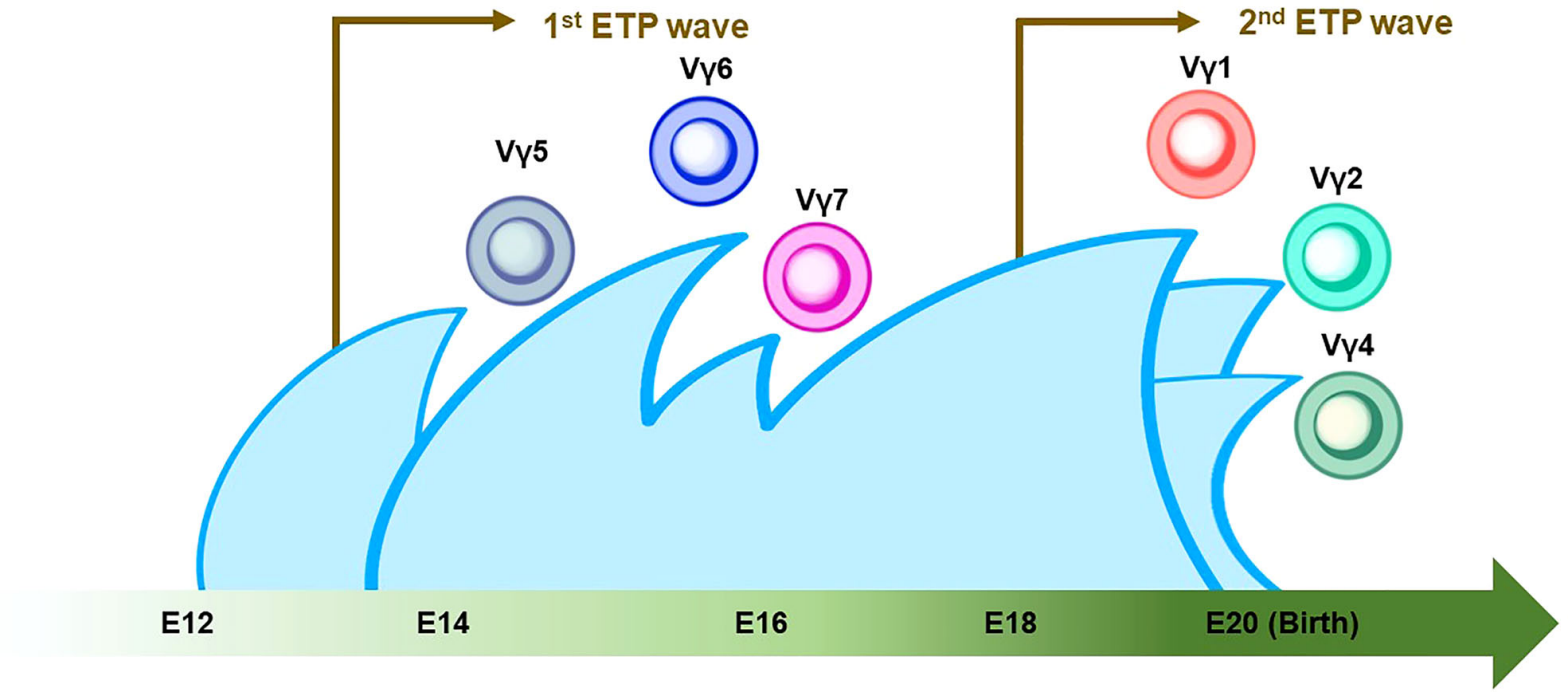
Thymic developmental waves of γδ T-cell subsets. The green arrow at the bottom indicates the developmental chronology of γδ T cells in the definitive wave. Vγ5^+^/Vγ6^+^/Vγ7^+^ γδ T cells emerge between E14 and E16, followed by the Vγ1^+^/Vγ2^+^/Vγ4^+^ γδ T cell around birth. Note that the first and second waves of early thymic progenitors (ETPs) come at E13 and E18 (indicated by brown arrows). The Vγ2^+^ γδ T cell shares a similar genomic signature with ILC2.

γδ T cells are the first T cells to develop in the thymus during fetal/neonatal life (79). γδ T cells go through the same developmental pathway as αβ T cells. TCR rearrangement starts from the γ and δ chains before the β chain during DN2a and DN2b developmental phases (80). γδ precursor cells express the TCRγδ/CD3 complex on the plasma membrane, like the pre-TCR, and initiates intracellular signaling pathways. This TCRγδ signal induces the process referred to as “γδ-selection” which triggers cell fate commitment of γδ T cells with the expression of functional TCRγδ (**Figure 2**) (78). Correspondingly, a previous study asked how Notch signaling influences developmental stages of γδ T cells and revealed that loss of Notch signaling led to a severe decline in γδ T-cell progenitor potential from DN2 cells, but not DN3 cells (81). In parallel, β-selection occurs in the DN3 stage, as mentioned above. Thus, these observations collectively suggest that the DN3 stage serves as an obligatory checkpoint at which the expression of the pre-T cell receptor (pre-TCR) or the γδ TCR determines the fate of the γδ or αβ cell lineages (78, 82, 83). According to the Vγ locus usage of reconstituted TCR, γδ T cells are subclassified into seven subtypes which are known to play biologically different roles in distinct anatomical areas. γδ T cells accomplish long-term persistence by characteristic abilities, including tissue residency and self-renewal capacity (82). From the ontological point of view, these subtypes are derived from two distinct hematopoietic progenitors, the E13 ETP which generates the first wave at E13 and the E18 ETP which does the second wave at E18 (84). After the first wave of the E13 ETP in mouse embryo, Vγ5^+^ appears on E15 and Vγ6^+^, and Vγ7^+^ follows. Subsequently, Vγ1^+^, Vγ2^+^, and Vγ4^+^ emerge through the second waves by E18 ETP (**Figure 3**) (82, 84–87). This observation suggests that two distinct hematopoietic progenitors give rise to different subsets of the γδ T-cell lineage, although they seem to undergo the same differentiation pathway.

## The rearrangement of the T-cell receptor locus in ILCs

While TCR gene expression is an indispensable feature of T-cell development, the seminal characteristics of ILCs include a lack of functional TCR expression. However, an unexpected discovery linked origins between T cells and ILCs in the research on the TCR gene in ILC progenitors. Previous studies used a Tcf7 reporter mouse system to cell-sort EILPs and ILCPs to propagate in cell culture. They showed that EILPs and ILCPs derived from adult BM were detected with high levels of unreconstituted TCR transcripts in RNA sequencing data. The observation suggested that EILPs and ILCPs express TCR transcripts in mRNA, although they lack TCR expression as proteins. These findings tempted the researchers to speculate a very close relationship between ILCs and T cells (32). Subsequently, an scRNA-sec analysis of cecum ILC subsets and lung ILC2 showed that transcripts of Cβ, a constant region of TCRβ, were expressed in all ILC subsets. In contrast, transcripts of other TCR chains, i.e., Cα, γ, and δ, were differentially expressed in each ILC subset. Genomic analysis revealed a genetic rearrangement of TCRγ but not in TCRβ in lung ILC2, which resembled the characteristics of mature Vγ2^+^ γδ T cells. Deletion of at least one allele of TCRδ and frameshift mutations in the Vγ2-Jγ1 rearrangement were frequently seen in ILC2 (88). Based on the fact including transcripts of the highly expressed constant region of the TCR and the non-sensical recombination of TCRγ and δ gene without VDJ recombination which should be completed to assemble the variable region of TCRγδ before the DN3 stage, it is suggested that ILC2 originated from the developmental phase between the DN1/ETP stage and DN3 stage in the γδ T-cell developmental pathway (78) (**Figure 2**). The scientific evidence indicative of the failure of reconstitution at the Vγ2-Jγ1 locus in tissue-resident ILC2 may be a sign of “failed” Vγ2^+^ γδ T-cell differentiation (**Figures 2** and **3**) (73). Based on the observations, including the presence of ILCs in the embryonic thymus and their dependence on early T cell transcription factors (89), it is noteworthy that ILC1 in human blood with and without the expression of EOMES, a master regulator of the development of ILC1 and NK cells, was reported to retain the expression of the T cell-related gene repertoire (90, 91). It was also asked whether EOMES^+/-^ ILC1s express TCR transcripts without surface TCR expression. The results showed that blood EOMES^+^ ILC1 express reconstituted αβ chains and that blood EOMES^-^ ILC1 rearranged all four TCR chains (90). Another report showed that splenic NK cells revealed a rearrangement of the TCRγ locus but not of TCRβ. On the other hand, unlike ILC2, NK cells have been reported to express rearranged TCRγ. Furthermore, a reconstituted TCRδ locus (Vδ4-Jδ1) was detected only in neonatal NK cells, suggesting that, as with ILC2, at least one allele may have been deleted due to failure of gene rearrangement at the TCRα/β locus (92).

Nonetheless, it has been disputed if the expression of TCR transcripts in ILCs might be due to experimental noises. A previous report showed that 7% of ILC1 upregulated the surface expression of TCRα/β following a 7-day culture and turned into the T cell-like phenotype (93). Another group demonstrated that a small proportion of ILC1 which transcriptionally expressed CD4, CD8A, and rearranged TCRα/β chains exhibited a partial clonal overlap with TCRα/β-rearranged T cells (90). These observations suggested that the current experimental design contaminated a small fraction of ILCs by misclassifying T cells, albeit with leading-edge technologies. Nonetheless, T-cell contaminants in an ILC population are a minor population. Therefore, ILCs are still likely to be derived from T-cell progenitors in the thymus (**Figures 2**, **3**), based on the evidence of the genetic signature and mRNA expression in the TCR locus in ILC. However, an improved methodology that controls the experimental noises may be necessary to conclude this question.

## Layered immunity models

**Figure 4:**
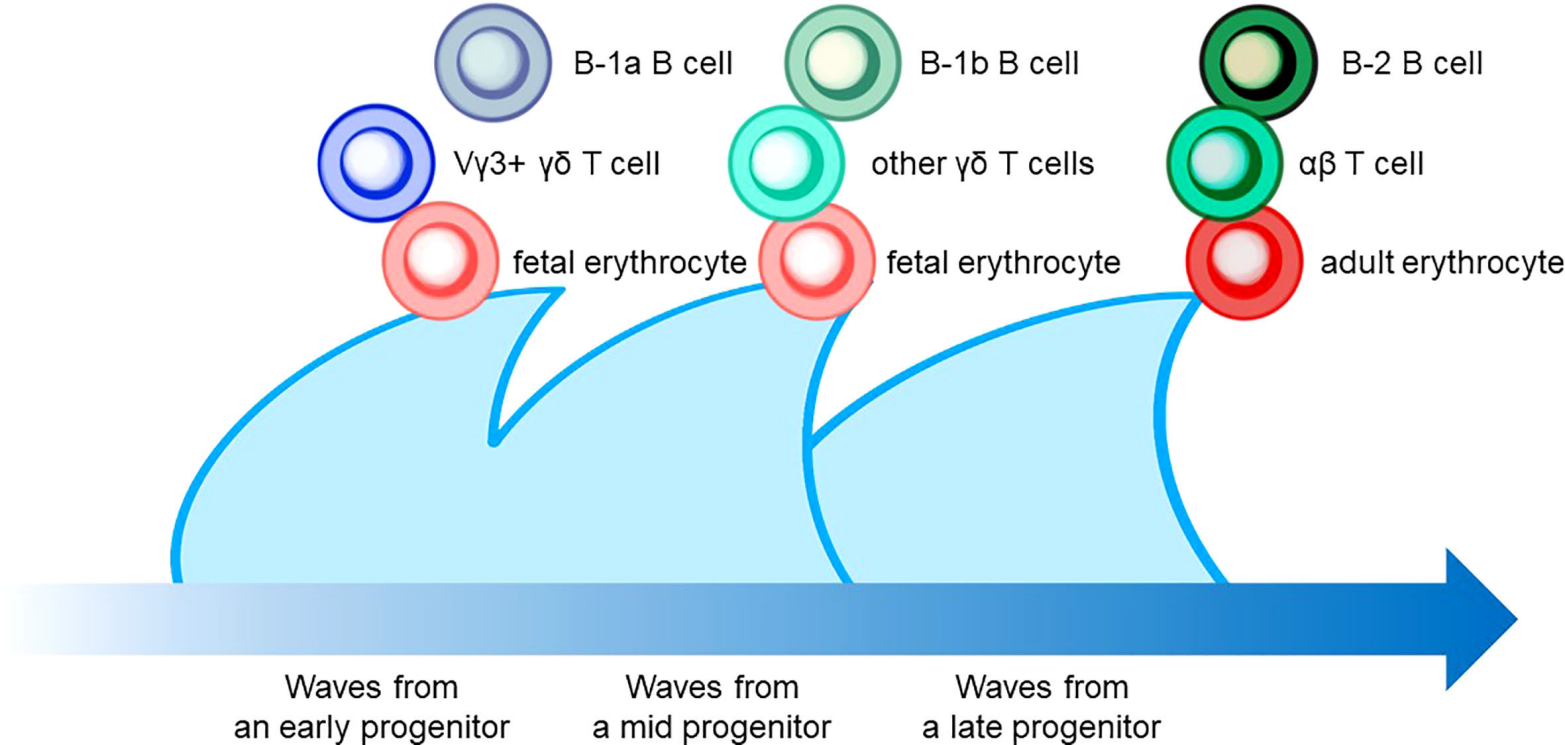
The layered immunity model which was proposed by Herzenberg et al. in 1992. A blue arrow at the bottom indicates the developmental chronology. In the concept, a hematopoietic progenitor during embryonic development generates the expansion of a unique cluster of blood and immune cells, which is described as a wave. Multiple waves generated by various progenitors compose multilayers of immune cell subsets, which collaboratively orchestrate the “organized” immunological function. Examples include B-cell, T-cell, and erythrocyte lineages that are derived from hematopoietic progenitors in different phases.

**Figure 5:**
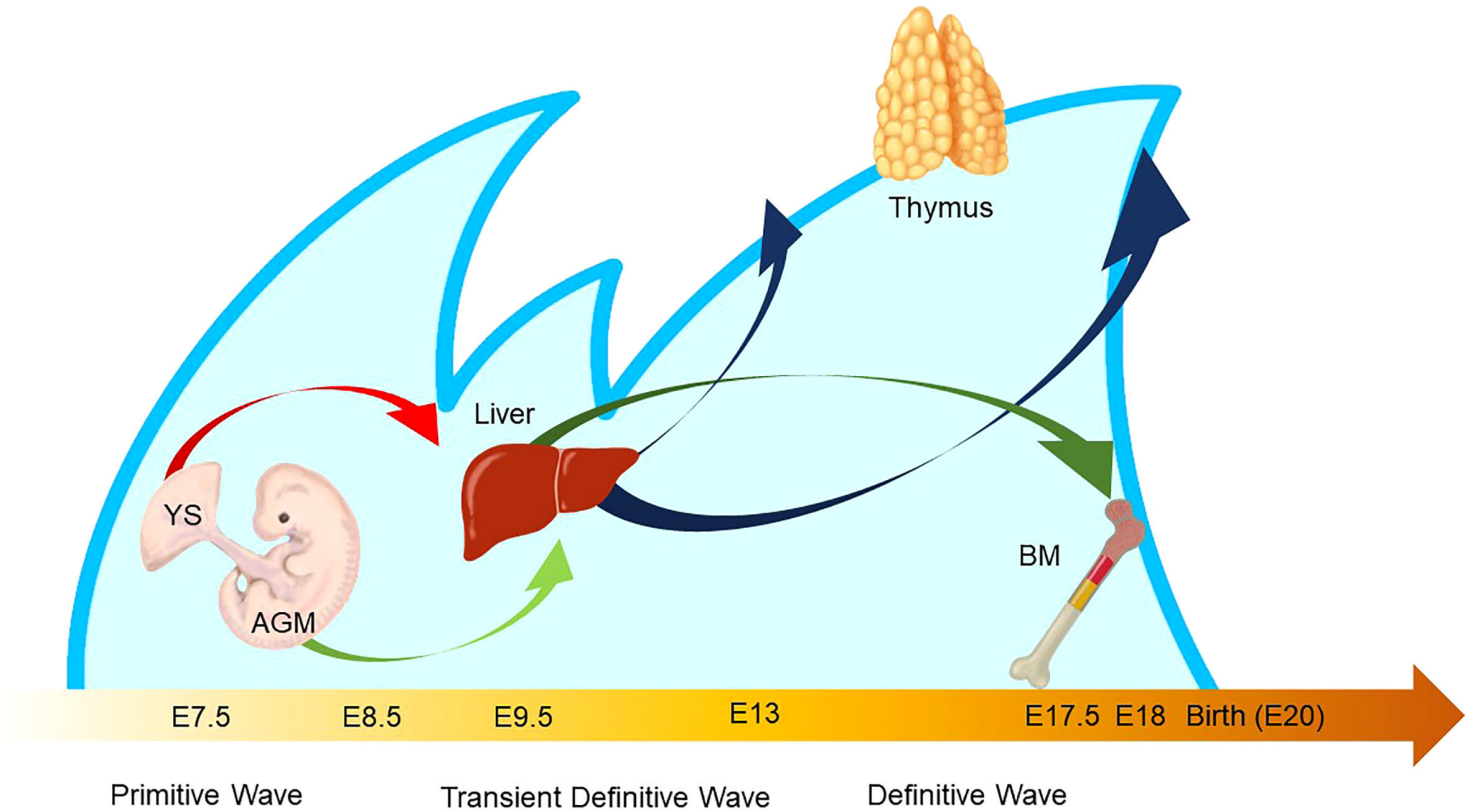
Waves of layered immunity during embryonic development in a mouse model. The blue waves indicate primitive, transient definitive, and definitive waves which start at E 7.5, E8.25, and E8.5, respectively. The blood islands in the yolk sac (YS) and aorta–gonad–mesonephros (AGM) independently give rise to their own hematopoietic progenitor cells. After the establishment of blood circulation, hematopoietic progenitors appear in the fetal liver at E 9.5. Then, the fetal liver turns into the main site for fetal hematopoiesis. Next, the waves of early thymic progenitors (ETPs) reach the thymus at E13 and E18. Subsequently, the hematopoietic progenitor cells are seeded into the bone marrow at E17.5.

The characteristic nature of ILC includes tissue residency and long-term persistence in specific anatomical sites, which resembles the characteristics of γδ T cells. Correspondingly, genomic sequence analysis showed similar patterns in the Vγ locus between ILC2 and γδ T cells. Other scientific evidence in the previous sections also suggested that a certain proportion of ILC may be derived from the embryonic thymus. On the other hand, intensive research revealed that postnatal BM produces at least some part of ILC. Thus, this raises the possibility that different progenitor cells with different natures in spatiotemporally distinct niches play a role as an ILC progenitor and collaboratively exert immune functions. This notion fits the concept of layered immunity, which was postulated in the research of innate-like lymphoid cells, such as γδ T cells and B-1a B cells (**Figure 4**) (94). Each progenitor cell during the embryonic phase produces a wave that expands an immune cluster with unique cell members as one layer in a stage- and site-specific manner. Thus, the more waves the progenitors produce, the more complex the immune network becomes with multiple layers of immune cells. The resultant layered immunity orchestrates the robust immune function. Research about the hematopoietic system in the embryo influenced the concept of layered immunity (95). On the one hand, the embryo requires mass production of differentiated red blood cells which supply oxygen to the rapidly growing body. On the other hand, undifferentiated hematopoietic stem cells need to be maintained throughout life. To achieve radically different goals, two types of fetal hematopoietic stem cells play roles in fetal development (**Figure 5**) (95). Early studies demonstrated that hematopoiesis occurs in two independent sites, YS and AGM. This led to a two-step model of the hematopoietic program: 1) a primitive wave with limited lineage potential independent of c-Myb, a hematopoietic master regulator gene, 2) a c-Myb-dependent definitive wave that gives rise to the first hematopoietic stem cells (HSCs) with long-term repopulation potential (96). The following studies which aimed to clarify the origin of HSCs discovered intermediate multipotent progenitors without long-term repopulation potential and assigned them as a transient definitive wave of hematopoiesis. Furthermore, recent scientific progress revealed that the concept is applicable to other cell types, such as tissue-resident macrophages (95). The primitive wave begins in the blood islands of the YS at E7.5 in mice (**Figure 5**) (65). During the primitive wave, precursor cells in the YS produce early hematopoietic progenitors (97). The first intra-embryonic hematopoietic progenitors are formed in AGM (65). The YS- and AGM-derived progenitors are locally confined until E8.5/E9.0. The establishment of blood circulation leads to seeding HSCs into the FL at E12.5 (65). The FL is known to be the major hematopoietic organ for erythropoiesis. The switch from the YS to the FL as the major site of hematopoiesis occurs when the primitive wave switches to the definitive wave (98). It is of note that two waves of hematopoietic progenitors which differentiate into ETPs and the following descendants, such as DN cells, αβ T cells, and γδ T cells in the thymus, emerge at E13 and E18 (**Figure 5**). In the approximately same phase, myelolymphoid progenitor cells are colonized in BM and become the primary site of hematopoiesis from E17.5 onward (**Figure 5**) (65). All myelolymphoid cells derived from AGM, YS, FL, and fetal BM disseminate to lymphoid and non-lymphoid tissues (63, 99, 100).

Recently, one group reported a seminal study to ask if ILC ontology may fit the layered immunity model (87). They investigated the difference in differentiation potentials toward innate immune cells between the first (E13) and second waves (E18) of ETPs in the developing thymus by using the mouse model with the fate-mapping technique. Inhibition of the first wave of ETP by anti-IL7Rα injection results in a significant reduction in thymic LTi cells, Vγ5^+^ γδ T cells, and mature mTEC expressing the autoimmune regulator (AIRE), suggesting that the first wave of the ETP contributes to the thymic development and establishment of immune tolerance (87). In addition, *in vitro* cell-culture assays demonstrated that the E13 ETP has a limited differentiation potential compared to the E18 ETP. Accordingly, the E13 ETP produced only LTi cells and T cells, while the E18 ETP produced B cells, ILC1/2/3s, and myeloid cells, indicating that the formation of the different immune layers is temporally regulated in the thymus. Correspondingly, data from a single-cell RNA sequence (scRNA-seq) exhibited the limited differentiation potential of E13 ETP which was transcriptionally primed toward the LTi cells and invariant T-cell profiles. In addition, the maturation of Vγ5^+^ γδT cells in thymic tissue, Vγ6^+^ γδ T cells in LNs, and medullary thymic epithelial cells (mTEC) in the thymus is dependent upon the first wave derived from the E13 ETP (87). Thus, these data can be interpreted as that E13 ETP mainly produces LTi cells, while E18 ETP gives rise to a wide range of ILCs. Furthermore, the data correspond to the previous studies that revealed that ILC2 harbored the genomic signature similar to Vγ2^+^ γδ T cells which emerge in the late stage of embryonic development (**Figures 3** and **5**).

The findings showed that E13 and E18 ETPs generated distinct ILC subsets that consist of unique cell members, which are also different from the ILC subset in adult BM. Thus, ILC ontogeny was consistent with the concept of layered immunity. Therefore, this suggests that many layers of ILC subsets synergistically exert a robust immunity function. Thus, it is necessary to comprehend how ILC subsets function and to which layer of the immune system the ILC subset belongs.

## Layered immunity suggests the origins of ILC subtypes

Despite accumulating findings of heterogeneity of ILCs, most previous studies have failed to reveal the biological significance of heterogeneity of ILC subsets. In this section, we introduce the ILC subtypes whose phenotypical differences might support the concept of layered immunity.

A mouse model of *Nippostrongylus brasiliensis* (*N. brasiliensis*) infection provided a clue to classify a subset of ILC2 with different responsiveness to epithelial alarmins, such as IL-25 (101). IL-25-responsive ILC2 was absent in the lung at a steady state but was found to accumulate in the lungs of mice 5 days after *N. brasiliensis* infection and disappear within 12 days. The different cellular kinetics delineate the existence of two subtypes of ILC2 in mouse: 1) the tissue-resident IL-33-reactive ILC2 which is referred to as natural ILC2 or nILC2 and 2) a circulating IL-25-responsive subtype which is termed as inflammatory ILC2 or iILC2. iILC2 harbors a high expression of KLRG1 and a low expression of CD90, whereas nILC2 harbors a low KLRG1 but high CD90 expression (102). As suggested by the responsiveness to specific cytokines, iILC2 preferentially expresses more IL-17rb, a subunit of an IL-25 receptor, while nILC2 does more ST2, a component of the IL-33 receptor on their surface (101, 103). Additionally, Arginase 1 (Arg1) was identified to discriminate between two subsets, although ILC2 and its progenitor cells shared the expression of Arg1, previously (18, 104, 105). In the experiment with Arg1 reporter mice, iILC2 hardly expressed Arg1 signals while nILC2 showed high Arg1 signals (103). On the other hand, BM-derived ILC precursors in the mouse do not express Arg1, while embryonic ILCPs express Arg1 (18). However, both IL-33-reactive nILC2 and IL-25-responsive iILC2 are known to coexist in the small intestine (106). One seminal research demonstrated that iILC2s migrate from the intestine to the lungs through the blood circulation under the influence of microbiota in the parabiosis model (106). Furthermore, they showed that the majority of iILC2 from the intestine turned into a tissue-resident nILC2-like phenotype, while the minor proportion was kept in circulation (106). Thus, it is suggested that the intestine can be a source of tissue-resident nILC2, at least in the lung and minor population of circulating iILC2. On the other hand, another research focused on the circulating iILC2 with the atopic dermatitis model, in combination with photoconvertible KikGR which change the fluorescent color from green to red by UV irradiation (107). They first characterized the circulating ILC2 in the draining LNs and the skin-resident ILC2s by scRNA-seq (107). Subsequently, ILC2 in the skin was tracked by KikGR after UV irradiation, which revealed that skin- (red) and LN-derived ILC2 (green) shared consistent gene expression patterns. These data suggest ILC2 in LN is KLRG+ iILC2 which goes back and forth between the skin and LN, while ILC2 in the skin consists of both circulating KLRG+ iILC2 and tissue-resident CD103+ ILC2 (107). In line with a previous finding that BM is the major source of KLRG^+^ iILC2 (108), this study suggested that iILC2 is a major population of circulating ILC2 which is derived from BM. Furthermore, recent studies revealed that the AP-1 superfamily transcription factor BATF provides the clue to comprehend the regulation mechanism of the ILC2 phenotype (103). BATF-deficient mice showed a significant reduction in migrating IL-25-responsive KLRG1^high^ ARG1^low^ iILC2 in the small intestine and complete loss of the population in the lung under *N. brasiliensis* infection. Thus, BAFT is considered to regulate the phenotypical change of nILC2 to iILC2. whose phenotypical differences might support the concept of layered immunity. Another group analyzed the BAFT function in nILC2 in an influenza virus infection model with BAFT-deficient mice (109). The group demonstrated that nILC2s lose their immune-protective properties and acquire pathogenic ILC3-like functions in the absence of BATF (109). These findings suggest that Arg1^+^ nILC2 retains a tissue-resident phenotype although nILC2 in the intestine and the lung can convert to the other phenotypes. On the other hand, it is also yet to be concluded if circulating iILC2 from BM can phenotypically convert into tissue-resident nILC2. A simple speculation is Arg1^+^ ILC2 is tissue-resident which can transiently convert into other subtypes, while KLRG1^+^ ILC2 is circulating.

**Figure 6:**
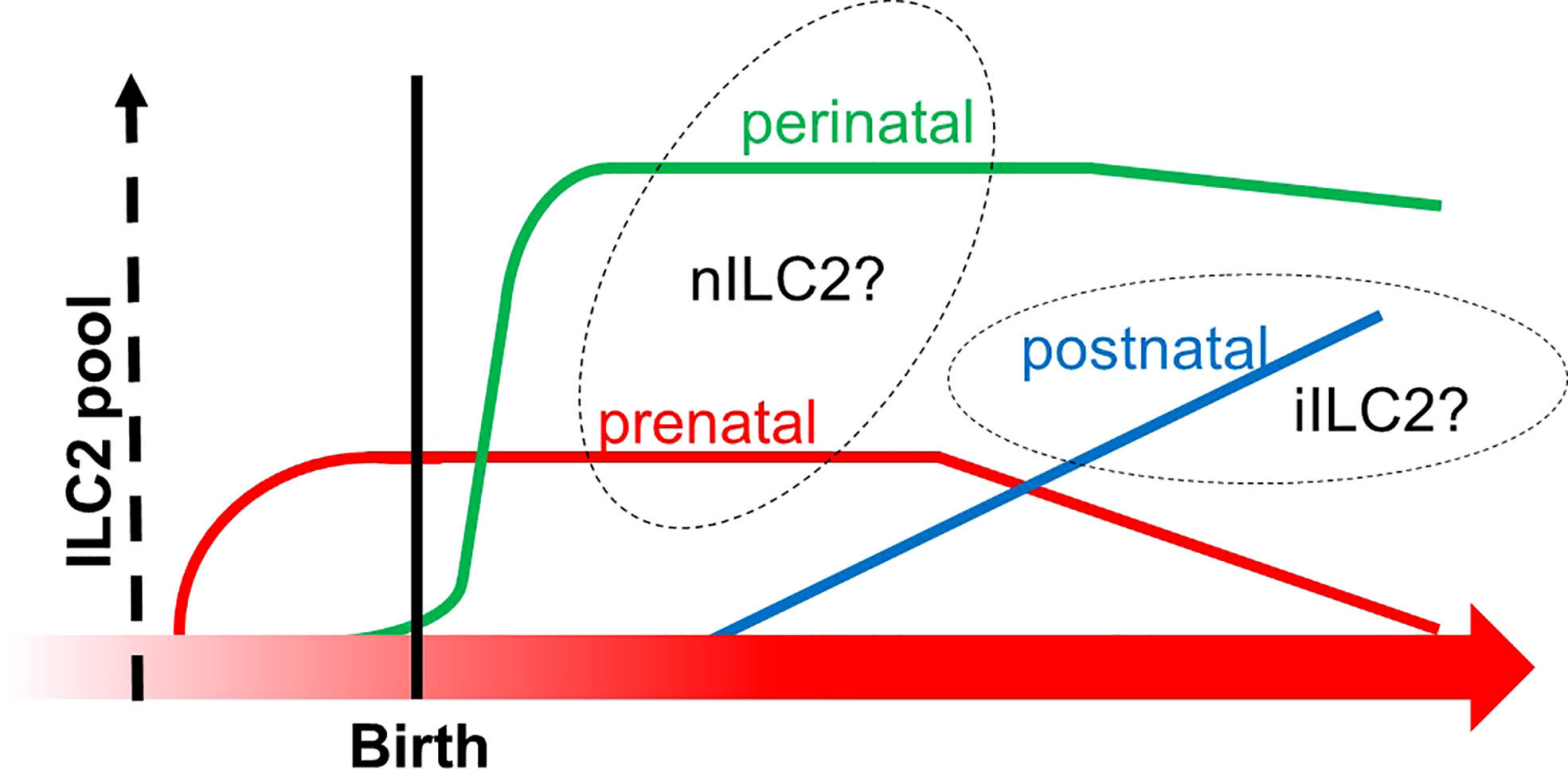
The transition of ILC2 pools from prenatal to postnatal phases. The X-axis indicates the developmental chronology, and the Y-axis shows the cellularity of the ILC2 pool. Three subgroups of ILC2 pools are depicted (prenatal in red, perinatal in green, and postnatal in blue). Prenatal and perinatal subgroups might be derived from the thymus since they increased before the bone marrow fully commenced hematopoiesis in the postnatal phase. This graph was created based on ref (110).

Another group directly asked if multilayered waves of ILC2 may contribute to innate immunity. The group performed experiments of time-controlled phylogenetic tracking of ILC2 by the fate-mapping approach combined with an Arg1- and Id2-driven ILC2 reporter mice (110). The study demonstrated that ILC2 emerged in multiple organs during late pregnancy (110), which corresponds to the timing of emergence of Vγ2^+^ γδ T cells that may share the same developmental pathway (**Figures 2** and **3**). During the postnatal phase, most of the peripheral ILC2 pool was generated by BM hematopoiesis, whose putative progenitors were not labeled in the Arg reporter system (**Figure 6**). On the other hand, the authors observed that Arg1^+^ ILC2 was seeded during the embryonic phase. Furthermore, prenatal and perinatal ILC2 were replaced throughout the tissue with age. Nonetheless, tissue-resident ILC2 was notably increased after helminth infection by the local proliferation but not due to *de novo* generation by BM hematopoiesis (110). These results indicated that ILC2 pools in the mouse are replenished in a temporally distinct manner and that ILC2 from postnatal BM behaves differently in response to external stimuli compared to embryonic ILC2. As mentioned above, the progenitor cells from AGM originate postnatal ILC2 from BM while E18 ETP produces ILC2 during the embryonic phase, as discussed in Section 5 (**Figure 5**) (87). In addition, BM is known as the major pool of KLRG1^+^ iILC2 (108). Thus, these observations fit the notion alongside layered immunity, that is, ILC2 progenitors from BM produce circulating KLRG1^+^ ILC2 postnatally, while ILC2 progenitors do tissue-resident Arg1^+^ ILC2 prenatally. This report further suggests that prenatal and perinatal ILC2 pools can to be distinguished since the cellular kinetics between them makes a clear contrast (**Figure 6**). Thus, it is also possible that another subset of innate lymphocytes in the embryonic thymus or FL could be a progenitor cells of prenatal ILC2 pools apart from the E18 ETP.

The ontogeny of LTi cells supports the concept of layered immunity. As described in Sections 2 and 5, FL and embryonic thymus serve as prenatal niches for LTi development. In addition, embryonic LTi cells were shown to be replaced by BM-derived LTi cells in adult mice (61). Notably, a research group asked if progenitors of LTi cells in FL are derived from YS or AGM (**Figure 1D**) (61). Since AGM but not YS expresses CXCR4 which exserts multiple functions, such as vascular, hematopoietic, and neural development (60, 111), they used the fate-mapping approach with the Cxcr4-CreErt2 mouse and demonstrated that progenitor cells of LTi cells were derived from the AGM region (**Figure 1D**) (61). Although the ontogeny of LTi cells can be well explained by the layered immunity model, intriguing biological questions remain: 1) if LTiPs in FL are the direct progenitor of E13 ETPs in the thymus or if they are distinct progenitors to give rise to different groups of LTi cells, 2) what the biological significance of BM-derived LTi cells is, and so on.

LTi cells were previously categorized as a member of a heterogeneous ILC3 group, based on dependency on the transcriptional differentiation program by RORγt in addition to overlap of phenotypical similarity. Nonetheless, ILC3 progenitors were shown to require PLZF for differentiation in both human and mice (112, 113). Nonetheless, LTi cells were still observed in the Zbtb16 knockout mice (43). Therefore, these data raised a possibility that LTi cells are non-ILC3 lineages (66). ILC3s consist of two major populations with or without natural cytotoxicity receptors (NCRs) that consisted of NKp46, NKp30, and NKp44 (114). ILC3 is known to promote intestinal immune and metabolic homeostasis. In fact, approximately 70% of ILCs are NCR^+^ ILC3 in the small intestinal tract, while 15% of them are NCR^−^ ILC3 (115, 116). NCR^+^ ILC3 primarily express IL-22, but less IL-17, while NCR^−^ ILC3 primarily express IL-17, but less IL-22 (117). *In vitro*, NCR^−^ ILC3 can switch to NCR^+^ ILC3 in the presence of IL-1β and IL-23 (118). NCR^−^ ILC3 includes another unique subpopulation termed LTi-like ILC3. As the name of LTi-like cells suggests the cell harbors the close gene expression profile to LTi cells, except that LTi-like cells express OX40L and CD30L (119). Nevertheless, LTi-like ILC3 is not capable of maintaining secondary lymphoid tissue, unlike LTi cells. On the other hand, it is demonstrated that LTi-like ILC3 is required for the postnatal development of tertiary lymphoid tissues, such as cryptopatches (114). Previous reports showed that LTi-like ILC3 (NCR^-^) was present in the fetal gut, while NCR^+^ ILC3s are largely absent (120, 121). Furthermore, they are known to be replenished postnatally by BM progenitors (120). However, the annotation of specific progenitor cells and a detailed differentiation pathway are yet to be investigated. As discussed in Section 2, PLZF^+^ ILCP produces ILC1, ILC2, and NCR^+^ ILC3, but not LTi-like ILC3, which suggests that PLZF^+^ ILCP have lost the capacity to generate LTi-like ILC3 progeny (43). A recent study annotated Arg1^+^ fetal ILCP (ftILCP) in the fetal intestine at E13 which generates ILC1/2/3 *in vitro*. Thus, these observations indicate that ILC3 is derived from various progenitors. Notably, ftILCP is apparently different from E18 ETP described in the previous section. Thus, it is of intrigue how the TCR transcript in ftILCP looks like since the modes of VDJ recombination in TCR could suggest the origin of ftILCP, as we observed the similarity between ILC2 from E18 ETP and γδ T cells.

NK cells have long been studied with the circulating NK cells in the blood since they were discovered in 1975 (1). The conventional NK cells are derived from BM driven by the expression of EOMES (91). On the other hand, tissue-resident NK cells are also known to exist in various organs and tissues (122, 123). Recent studies revealed that the cells derived from E8.5 YS in mice harbored a potential to differentiate NK cells (122, 124). Moreover, ILC1 in the liver, which some researchers regard as tissue-resident NK cells, was intensively studied to clarify if the origins of ILCs and NK cells are different or not. One seminal study with the PLZF-fate mapping mouse system demonstrated that tissue-resident NK in the liver was not derived from EILP or CHILP, but from unique PLZF-expressing ILCPs (113). The research group showed that the developmental pathway of tissue-resident NK cells in the liver was similar to that of conventional NK cells, although the progenitor cells were distinct (113, 122). These findings again tempt us to speculate the existence of at least two subtypes of NK cells: tissue-resident NK cells that harbor an ILC1-like phenotype and are possibly derived from YS *via* FL, and circulating, conventional NK cells that are derived from BM.

The contrastive examples between tissue-resident and circulating subsets of ILCs imply that tissue-resident ILCs are seeded during prenatal to perinatal phases while circulating ILCs are derived from postnatal BM. Further study is needed to clarify if the concept of the layered immunity model can be generalized to much broader cell types by the characteristic feature of tissue residency.

## Concluding remarks

Here, we introduced the scientific evidence for different sites of ILC ontogeny, the similarity of ILC to γδ T cells, and the possible classification of ILC subtypes based on the layered immunity. This notion may enable the delineation of the accurate figures of elusive ILCs. Although it could be oversimplified, ILCs might be described in light of the concept of layered immunity as follows. 1) Tissue-resident ILCs are primarily seeded during the embryonic phase. 2) At least a part of tissue-resident ILC2 undergo the same differentiation pathway as γδ T cells and are branched from DN cells in the thymus. 3) Circulating ILCs are derived from BM and replace tissue-resident subtypes in the various tissue postnatally. 4) Multipotent and unipotent ILCPs that give rise to circulating ILC can stochastically propagate into any subtypes of ILCs. 5) Circulating ILCPs provide an “on-demand” supply of ILC subsets in the inflammatory sites.

It has just begun that the layered immune concept has been applied to account for some parts of the biological significance of ILCs. However, the concept of layered immunity showed the great potential to disentangle the daunting complexity of heterogeneity of ILCs. Therefore, further studies for ILC-biology in light of layered immunity are necessary.

## Author contributions

TK wrote the first draft of this manuscript. GE, TN, and KK contributed to editing the manuscript. All authors contributed to the article and approved the submitted version.

## Funding

Takeda Sciences Foundation, Grants-in-aid for Scientific Research (21K16227, 20K08649, and 21K16227).

## Conflict of interest

The authors declare that the research was conducted in the absence of any commercial or financial relationships that could be construed as a potential conflict of interest.

## Publisher’s note

All claims expressed in this article are solely those of the authors and do not necessarily represent those of their affiliated organizations, or those of the publisher, the editors and the reviewers. Any product that may be evaluated in this article, or claim that may be made by its manufacturer, is not guaranteed or endorsed by the publisher.
